# Early and late onset sepsis in late preterm infants

**DOI:** 10.1186/1824-7288-40-S2-A23

**Published:** 2014-10-09

**Authors:** Fabio Natale, Bianca Bizzarri, Veronica Cardi, Mario De Curtis

**Affiliations:** 1 Department of Pediatrics and Child Neuropsychiatry, “Sapienza” University of Rome, Rome, Italy

## 

Preterm birth is increasing worldwide. Late preterm infants (from 34+0 to 36+6 weeks of gestation) comprise more than 70% of premature infants and this percentage has been increasing in recent years. These infants, though resembling normal term infants, due to an incomplete maturational process, suffers higher percentage of mortality (from 0,5 to 1,5/1000 vs 0,2/1000 live births) and morbidity (from 17% to 34% vs 14%) if compared to term ones [1].

Late preterm infants are inherently prone to develop sepsis: 1) Preterm labor and preterm premature rupture of membranes, together accounting for the 80% of the causes of preterm delivery [2], are well known risk factor for early onset sepsis (EOS); in fact, intrapartum antibiotic prophylaxis is widely recommended to prevent group B streptococcal EOS in women undergoing preterm delivery (risk factors approach), and sepsis work-up is frequently performed in late preterm infants [1]. 2) Some degree of immaturity of both innate and adaptive immunity makes late preterm infants at increased risk to develop sepsis [3]. 3) Increase rate of morbidity (respiratory problems, needs for reanimation in delivery room, hyperbilirubinemia, hypoglicemia, and feeding problems) exposes these infants to prolonged hospitalization and invasive procedures favouring nosocomial infections to occur [1].

Mc Intire et al. reported an incidence of culture proven sepsis from 2 to 5 times higher in late preterm infants (the incidence was inversely related to the gestational age) when compared with 39 weeks gestation infants [1]. Neonatal infections in late preterm infants (including culture-proven and suspected sepsis) were 5,2 times more common in a population-based study lead by Khashu et al [4]. Cohen-Wolkowiez et al. [5], were the only to specifically address the issue of the incidence of EOS and late onset sepsis (LOS) in late preterm infants. An observational cohort study comprising more than 100,000 late preterm infants admitted to 248 neonatal intensive care units from 1996 to 2007 revealed a cumulative incidence of 4,42/1000 admissions for EOS and 6,3/1000 admissions for LOS. Gram-positive organisms accounted for the majority (66,4%) of EOS but mortality (1,3% of all EOS episodes) was mainly due to Gram-negative sepsis (19,1% vs 1,1%). Of the neonates suffering LOS, 7% died. Infants with LOS were three times more likely to die than infants without sepsis.

Prevention of sepsis in late preterm infants may be best accomplished through maternal antibiotic prophylaxis (when indicated), and avoiding non-indicated late preterm deliveries (17%-25% of all cases) [2,6].
